# Prostaglandin E2 induces immediate migraine-like attack in migraine patients without aura

**DOI:** 10.1186/1129-2377-14-S1-P113

**Published:** 2013-02-21

**Authors:** M Antonova, T Wienecke, J Olesen, M Ashina

**Affiliations:** 1 Danish Headache Center, Denmark

## Background

Prostaglandin E2 (PGE2) has been suggested to play an important role in the pathogenesis of migraine [1]. In the present experiment we investigated if an intravenous infusion of PGE2 would induce migraine-like attacks in patients with migraine.

## Methods

Twelve patients with migraine without aura were randomly allocated to receive 0.4 ìg/kg/min PGE2 (Prostin®E2, dinoprostone) or placebo over 25 min in a two-way, cross-over study. Headache intensity was recorded on a verbal rating scale, middle cerebral artery blood flow velocity (VMCA) was measured by Transcranial Doppler (TCD) and diameter of superficial temporal artery (STA) was obtained by c-series scan (Dermascan C).

## Results

In total 9 migraine patients (75%) experienced migraine-like attacks after PGE2 compared to none after placebo (P = 0.004). Seven out of 9 (58 %) patients reported the migraine-like attacks during the immediate phase (0-90 min) (P = 0.016). Only two patients experienced the delayed migraine-like attacks several hours after the PGE2 infusion stop (P = 0.500). The VMCA decreased during the PGE2 infusion (P = 0.005) but there was no significant dilatation of the STA (P = 0.850).

## Conclusion

The migraine-like attacks during, and immediately after, the PGE2 infusion contrast with those found in the previous provocation studies, where the other pharmacological compounds triggered the delayed migraine-like attacks several hours after the infusion. We suggest that PGE2 may be one of the important final products involved in the generation of migraine attacks.
